# Molecular dynamics investigation of the interaction between *Colletotrichum capsici* cutinase and berberine suggested a mechanism for reduced enzyme activity

**DOI:** 10.1371/journal.pone.0247236

**Published:** 2021-02-19

**Authors:** Ying Li, Jinqing Wei, Huizhen Yang, Jing Dai, Xizhen Ge

**Affiliations:** 1 Beijing Key Laboratory of Biomass Waste Resource Utilization, College of Biochemical Engineering, Beijing Union University, Beijing, China; 2 Beijing Aerospace Petrochemical EC&EP Technology Co., Ltd, Beijing, China; GC University Faisalabad, PAKISTAN

## Abstract

Berberine is a promising botanical pesticide against fungal plant pathogens. However, whether berberine inhibits the invasion of fungal pathogen across plant surface remains unclear. Here we demonstrated that the enzyme activities of purified cutinase from fungal pathogen *Colletotrichum capsici* were partially inhibited in presence of berberine toward different substrates. Molecular dynamics simulation results suggested the rigidity of cutinase was decreased with berberine added into the system. Interestingly, aggregations of berberine to the catalytic center of cutinase were observed, and stronger hydrophobic interactions were detected between key residue His 208 and berberine with concentrations of berberine increased. More importantly, this hydrophobic interaction conferred conformational change of the imidazole ring of His 208, which swung out of the catalytic center to an inactive mode. In summary, we provided the molecular mechanism of the effect of berberine on cutinase from *C*. *capsici*.

## Introduction

Plant diseases caused by fungal pathogens are serious problems worldwide [1]. Among all the pathogenic factors of fungal pathogens, cutinase is the most important one that plays a role by invasion of hydrophobic layer in plant surfaces [2]. Strong evidences exist that cutinase released during the first step of plant invasion are involved in the penetration of this layer and thus, constitute a crucial factor of pathogenicity [3]. Up to now, various studies had revealed the crucial role of cutinase in pathogenicity presented for host-pathogen system [4]. At the same time, cutinase-deficient strains of several fungi were nonpathogenic on intact surfaces, but supplementation of purified cutinase restored their pathogenicity [5]. All these results provided several lines of evidence that cutinase are involved in degradation of plant cuticle and the enzyme activity of cutinase is an essential factor of fungal pathogenicity. Thus, reduction of cutinase activity of fungal pathogens is important for preventing fungal pathogen invasion.

Our previous studies demonstrated that berberine is a promising compound to inhibit growth of *Monilinia fructicola* [6, 7], a fungal pathogen responsible for peach brown rot. The EC_50_ of berberine against *M*. *fructicola* is as low as 4.5 ug/ml, showing great potential in preventing this plant disease. Interestingly, activity of cutinase secreted by *M*. *fructicola* was reduced after cultivation with berberine [6], providing a possible anti-invasion mechanism of berberine. However, the inhibitory mechanism of berberine against cutinase activity remains unclear. Indeed, there are several pesticides or compounds function as inhibiting the enzyme activity of cutinase from fungal pathogens for plant disease control, such as benomyl, (R)-1,2-dibutyl-carbamoylglycero-3-O-p-nitrophenylbutyl-phosphonate, and diisopropylfluorophosphate [8, 9]. Nevertheless, few compounds were successfully applied in large scale due to their high cost or potential pollutions to the environment. Thus, as an environmental friendly compound, berberine is promising to prevent pathogens invasion by inhibiting cutinase enzyme activity if berberine indeed affects enzyme activity of purified cutinase from fungal pathogens.

The natural function of cutinase is to catalyze the hydrolysis of ester bonds in cutin, which is the main component in cuticle found in higher plants. Due to the special ability to degrading polyesters, cutinase had been widely applied in detergents and textile industry [10]. The structures and enzyme activities of cutinase from different species had been extensively characterized, providing powerful background for mechanism investigation. The catalytic triad of cutinase was consisted by Ser 105, His 173 and Asp 160 [11]. The loops near His 173 functions as a lid guarding the catalytic triad, while the lid is not large enough in keeping water-soluble substrates out of the active site. Thus, some water soluble compounds such as sodium dodecyl sulfate (SDS) or ethylene glycol, had been studied their cutinase inhibitory effects simultaneously by wet-experiments and molecular dynamics (MD) [12, 13]. The cutinase inhibition by SDS is involved the nucleation of aggregates of SDS molecules on hydrophobic patches on the cutinase surface, leading to the blockage of the entrance to the catalytic triad. These studies provided a possible mechanism of cutinase inhibitory effect of berberine since berberine has two hydrophobic groups in each end of its molecule.

On the basis of the studies mentioned above, researches were conducted on the inhibitory effect of berberine against cutinase from fungal plant pathogen *Colletotrichum capsici*. Our results indicated that berberine affected the enzyme activity of cutinase toward different substrates. Meanwhile, mechanisms of this inhibitory effect were revealed by MD analysis of cutinase-berberine system. Our data addressed the aggregation of berberine to the catalytic center of cutinase in different concentrations of berberine. Moreover, the hydrophobic interaction between berberine and histidine in the catalytic center conferred its significant conformational change to an inactive mode. On the basis of our findings, our data suggested the inhibitory mechanism of berberine against cutinase and implied a novel role of berberine in preventing fungal plant disease.

## Materials and methods

### Chemicals

Recombinant cutinase from plant pathogen *C*. *capsici* (UniProt: P10951) was purchased from Biomatik (RPC26304). Then cutinase was dissolved in distilled water contains 15% of glycerol to reach a final concentration at 1 μg/ml. Substrates used for determination of cutinase activity were products of Sigma (Tributyrin, W222305; Glyceryl trioctanoate, T9126; Glyceryl tribenzoate, 389188; Isopropyl myristate, 172472), representing different kinds of esters in plant surface. PBS buffer was also a product of sigma (P4417) to provide an environment for enzyme activity assay.

### Determination of cutinase activity

Enzyme activity of cutinase was determined according to the reference [14]. Briefly, 0.1 ml of cutinase solution was added to 2 ml of PBS-berberine solution to reach the targeted concentrations of berberine. After cultivation of cutinase-berberine mixer at 30°C for 1 h, different substrate emulsion was added into the solution for 20 min incubation at 30°C. Finally an ultraviolet spectrophotometer (2600 UV-VIS; UNICO, USA) was used to test the optical density (OD_630_) before and after incubation. One unit of cutinase enzyme activity was defined as a 0.001 OD decrease in the absorbance per minute at 630 nm. Substrate emulsions were prepared by emulsifying substrate in 25 mM potassium phosphate buffer (pH 8.0) and 0.5% (w/v) gum arabic for 2 min at maximum speed in a Waring blender.

### Molecular Dynamics (MD) analysis

The structure of cutinase were generated based on the characterized structures of cutinase from RSC PDB bank (PDB: 1CEX, 3DCN, 3EF3, 4OYL and 4OYY). Homology modeling was performed in YASARA structure with the macro ‘hm_built’. All molecular dynamics simulations were also conducted in YASARA structure using AMBER14 force field. Cutinase was embedded in a simulation cell with the volume of 70 A*70 A*70 A. Different concentrations of berberine were added into the simulation box according to the concentrations used in wet-experiments. Then the system was cleaned and certain sodium irons were added into the simulation box to reach neutral. Temperature control was imposed using a Berendsen-type algorithm, and they were in accordance with the experimental condition at 30°C. For energy minimization, the steepest descent method was used in the first 5000 steps and a conjugate gradient method in the last 5000 steps. After energy minimization, each system was gradually heated to the experimental temperature. Each MD run was performed for 30 ns using a time step of 0.1 ns.

### MD data analysis

The data of MD were analyzed by the marco ‘MD_analyze’ in YASARA. Root mean square deviation (RMSD) between structures following least-squares fitting to a referenced initial structure was computed. Root mean square fluctuations (RMSF) of residues were computed to check the flexibility of each residue. The distance between catalytic center and berberine was determined by the distance between the geometric center of the key residues and berberine molecule. Hydrophobic interaction was defined if the distance between the two hydrophobic groups was lower than 5 Å. The data were plot in Python matplotlib module. Snapshots in this work were visualized in Pymol.

## Results

### Berberine affected cutinase activity toward different substrates

**Fig 1:**
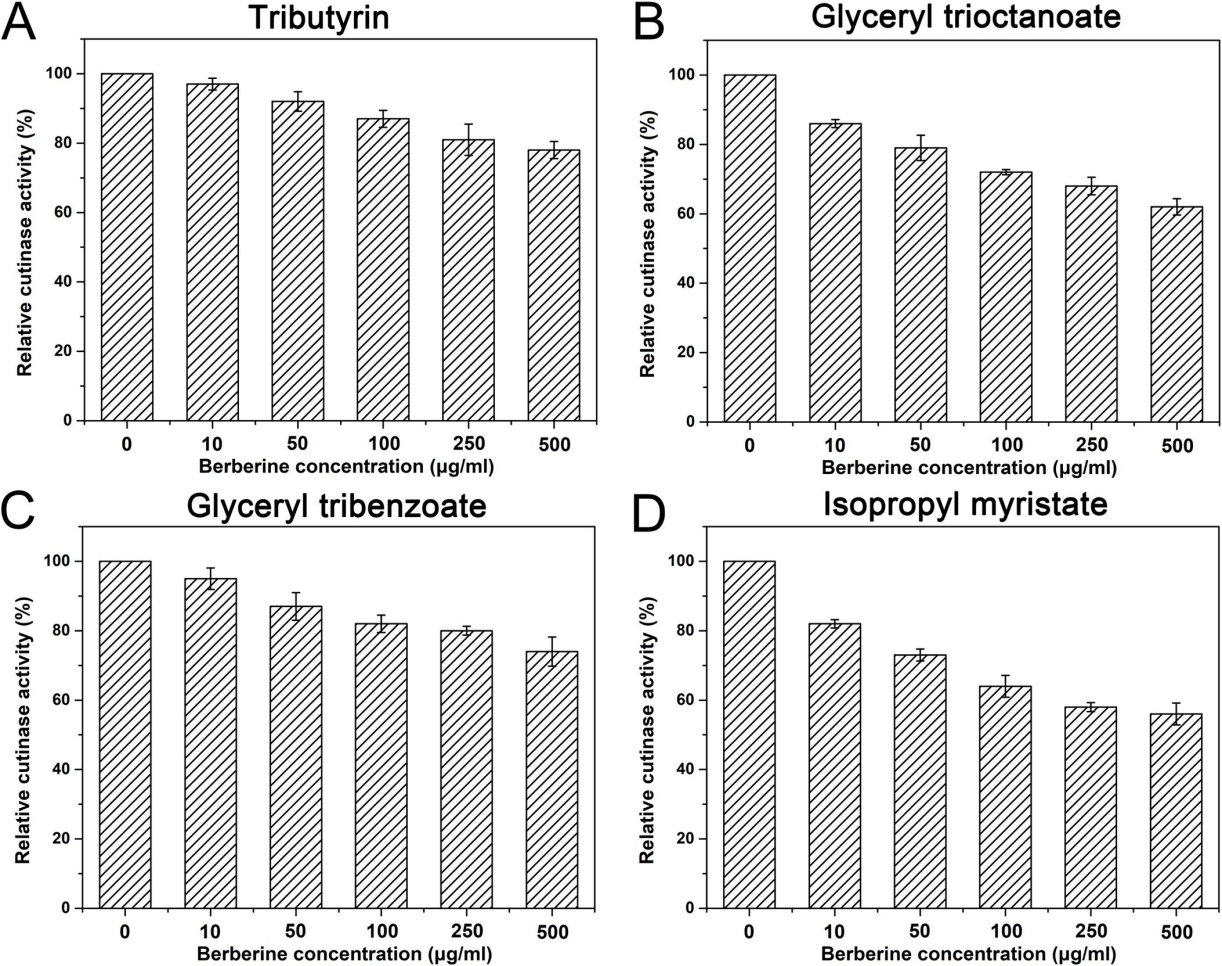
Berberine conferred reduced cutinase activity toward different substrates. Cutinase was first incubated with different concentrations of berberine for 1 h followed by determination of enzyme activities toward different kinds of polyesters. The enzyme activities without berberine were set as control. Error bar represented the standard error of three independent experiments.

The inhibitory effects of berberine on cutinase from *C*. *capsici* were tested toward different substrates (Fig 1). For all the substrates tested, enzyme activities of cutinase were significantly reduced with the increased concentrations of berberine. However, the relative enzyme activities were reduced to different levels toward different substrates. For short chain substrate tributyrin, berberine conferred slight decrease of relative activity. Even when 500 μg/ml of berberine is presented in the solution, 78% of the total activity was detected. Similarly, berberine conferred slightly reduced cutinase activity toward the aromatic ester glyceryl tribenzoate, and 74% of total was reserved compared to the control. In contrast, the presence of berberine resulted in larger reduced enzyme activities for longer chain substrates glyceryl trioctanoate and isopropyl myristate. Even low concentrations of berberine (10 μg/ml) resulted in 14% and 18% decrease of the total cutinase enzyme activities toward these two substrates. With 500 μg/ml berberine added into the system, the relative enzyme activities fall to 62% and 56% toward these two substrates. In total, berberine affected cutinase enzyme activity toward different substrates, and higher inhibitory effects were observed toward longer chain substrates.

### Berberine decreased rigidity of cutinase

**Fig 2:**
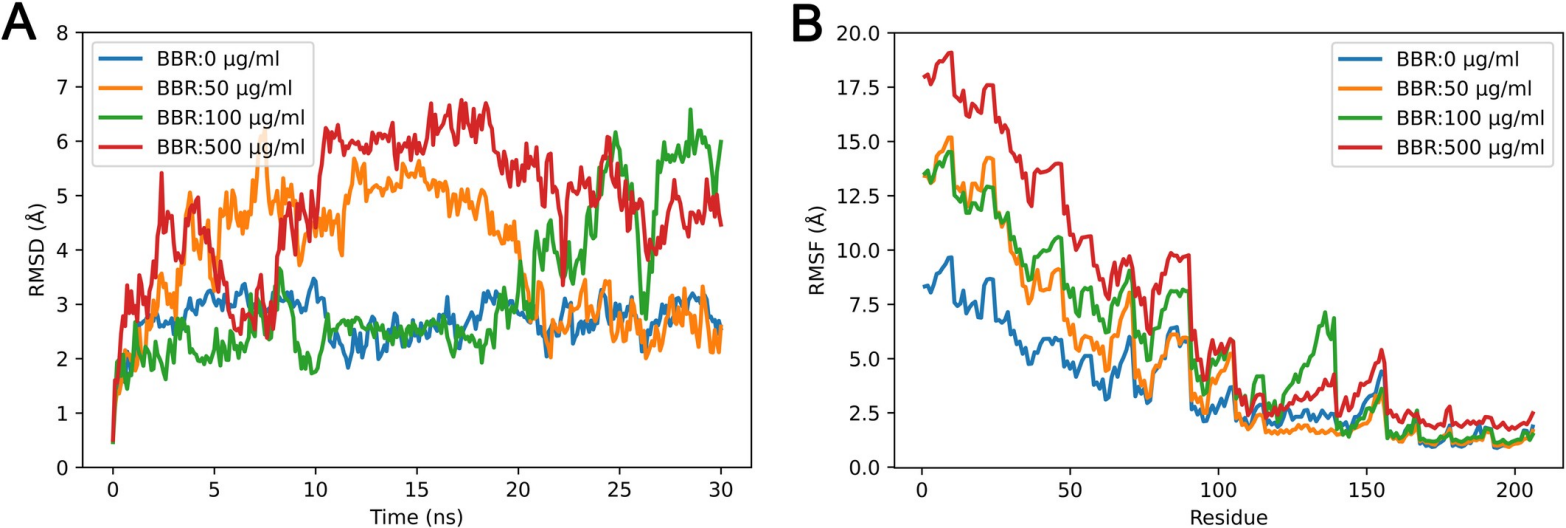
Berberine conferred reduced rigidity of cutinase during simulations. RMSDs (A) and RMSFs (B) were calculated in each simulation with different berberine concentrations. BBR: berberine.

MD simulations of cutinase were conducted with different concentrations of berberine in the system, and the RMSD and RMSF values were calculated throughout each of the 30 ns simulation (Fig 2). The backbone RMSD value of the control reached a plateau after 5 ns. From 10 ns to the end of simulation, RMSD of the control was kept stable between 2 Å and 3 Å. In contrast, the addition of berberine significantly affected RMSD values. For berberine concentration at 50 μg/ml, RMSD was largely increased from 5 ns to 20 ns, but it decreased similar to the control in the last 10 ns. In compare, when the concentrations of berberine were higher, the RMSD values were mostly affected in the second half of simulations. At the same time, different RMSF values in presence of berberine also indicated the reduced rigidity of cutinase. With the increased concentrations of berberine, RMSF values were significantly increased. Notably, the RMSF values of Ser 140 in the catalytic center were rapidly promoted with increased concentrations of berberine in the system. The total RMSD and RMSF values suggested that the presence of berberine decreased the rigidity of cutinase, which is suggested to contribute to its reduced activity [15].

### Aggregation of berberine to the catalytic center

**Fig 3:**
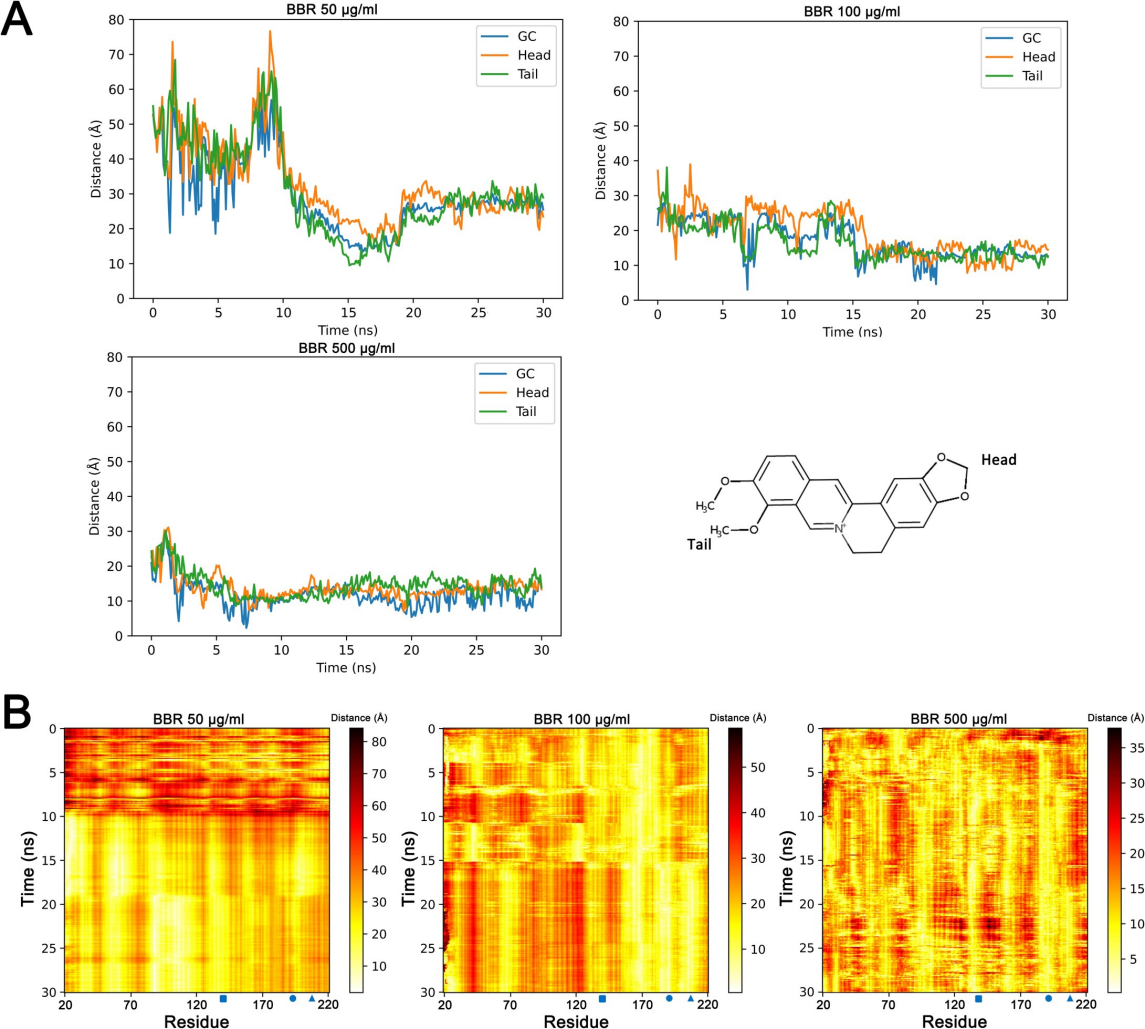
Aggregation of berberine to the catalytic center of cutinase. Distance between the cutinase catalytic center and berberine molecule (Head, tail and geometric center) was calculated throughout each simulation (A). Distances between each residue of cutinase and berberine molecule were computed throughout each simulation (B). GC: geometric center. Blue square: Ser 140. Blue circle: Asp 195. Blue triangle: His 208. BBR: berberine.

To illustrate the relationship between berberine and the catalytic center of cutinase, the emergence of berberine near the catalytic center (<5 Å) was computed in each simulation (S1 Fig). When the concentration of berberine was 50 μg/ml, single berberine molecule occurred near the catalytic center at several time points. With increased berberine concentrations, much higher possibilities were detected. At 500 μg/ml, two or three berberine molecules were detected close to the catalytic center for most of the snapshots. The distances of berberine to the catalytic center were calculated throughout the simulations (Fig 3). In all the simulations, the distances of berberine to the catalytic center became closer than initial. With the increased concentrations of berberine, these distances were much closer at the end of simulations. Since aggregations were observed in each group, the postures of berberine were determined when aggregating to the catalytic center. Surprisingly, the results indicated that the head and tail of berberine molecule shared similar possibilities in aggregating to the catalytic center in each berberine concentration (Fig 3A). Next, the distances of berberine with each residue of cutinase were measured throughout the simulations (Fig 3B). The distances of berberine with the three residues of catalytic center became closer with simulations carried on, and stayed at close positions at the end of each simulation. Except for the three key residues, the distances of berberine with Thr 169, Pro 95 and Ile 68 were also decreased throughout the simulations, and these three residues were all located near the catalytic center. Together, these data suggested the aggregations of berberine to the catalytic center at different concentrations.

### Hydrophobic interactions conferred conformational change of cutinase

**Fig 4:**
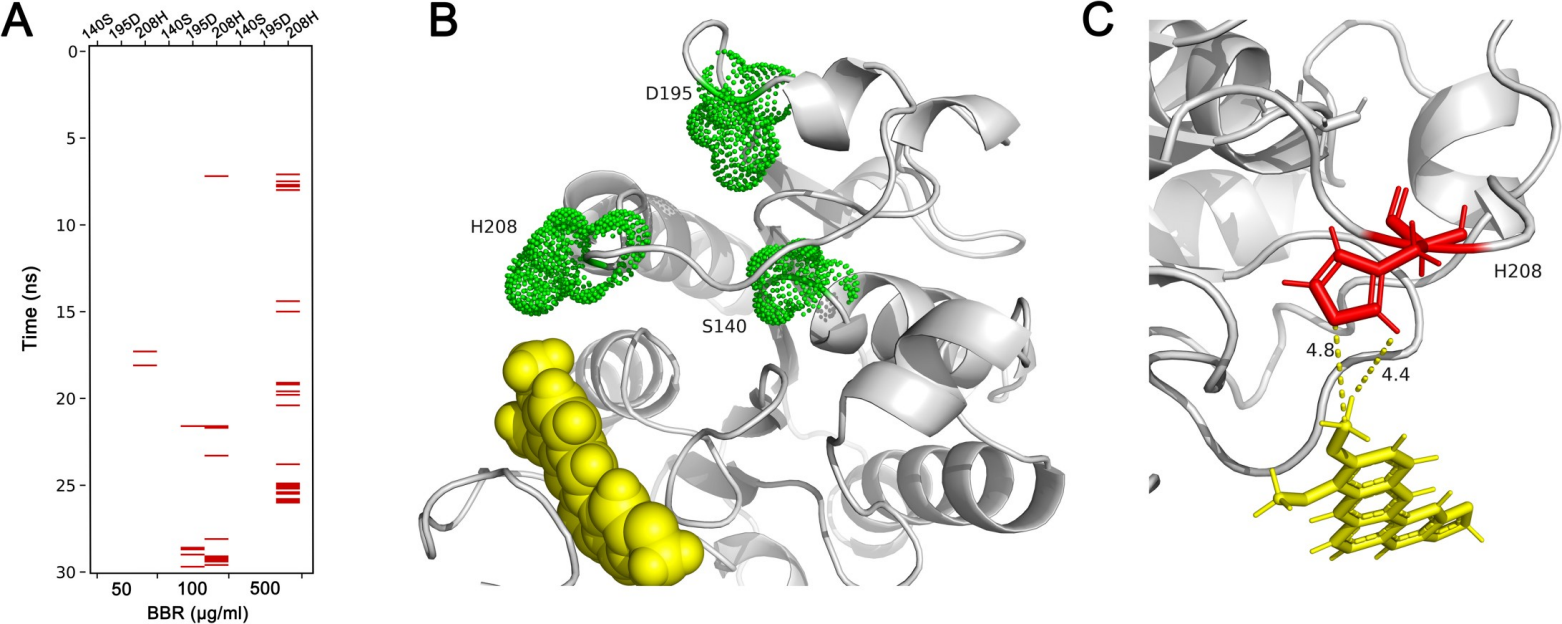
Hydrophobic interactions between key residues and berberine. Hydrophobic interactions of key residues and berberine molecules were detected throughout each simulation (A). A snapshot of hydrophobic interaction between His 208 and berberine was obtained at 18.3 ns at 50 μg/ml berberine concentration (B). A close up view of the hydrophobic interaction (C) show the distance between imidazole ring of His 208 and methyl group of berberine was lower than 5 Å. BBR: berberine.

Since the aggregations of berberine to catalytic center were observed in these simulations, the interactions between berberine and cutinase were researched, and the results indicated hydrophobic interactions between them (Fig 4). High possibilities of hydrophobic interactions between berberine and His 208 were identified as the simulations carried on (Fig 4A). With the increased concentrations of berberine, hydrophobic interaction possibilities became higher. Meanwhile, there are also a small part of hydrophobic interactions between berberine and Asp 195 when the concentration of berberine was 100 μg/ml, while it was not observed at higher or lower berberine concentrations. In contrast, no hydrophobic interaction was detected between berberine and Ser 140 in all simulations since it is a polar residue. A snapshot was obtained at 18.3 ns with 50 μg/ml berberine in the simulation system (Fig 4B and 4C), in which a strong hydrophobic interaction was observed between the imidazole ring of His 208 and methyl group of berberine. The distances between the atoms of the two groups were both closer than 5 Å. Since the head and tail of berberine molecule had similar possibilities in the aggregation analysis, the other representative snapshots were visualized and displayed in S2 Fig, suggesting strong interactions between berberine and His 208 since different atoms of berberine were found to interact with His 208.

**Fig 5:**
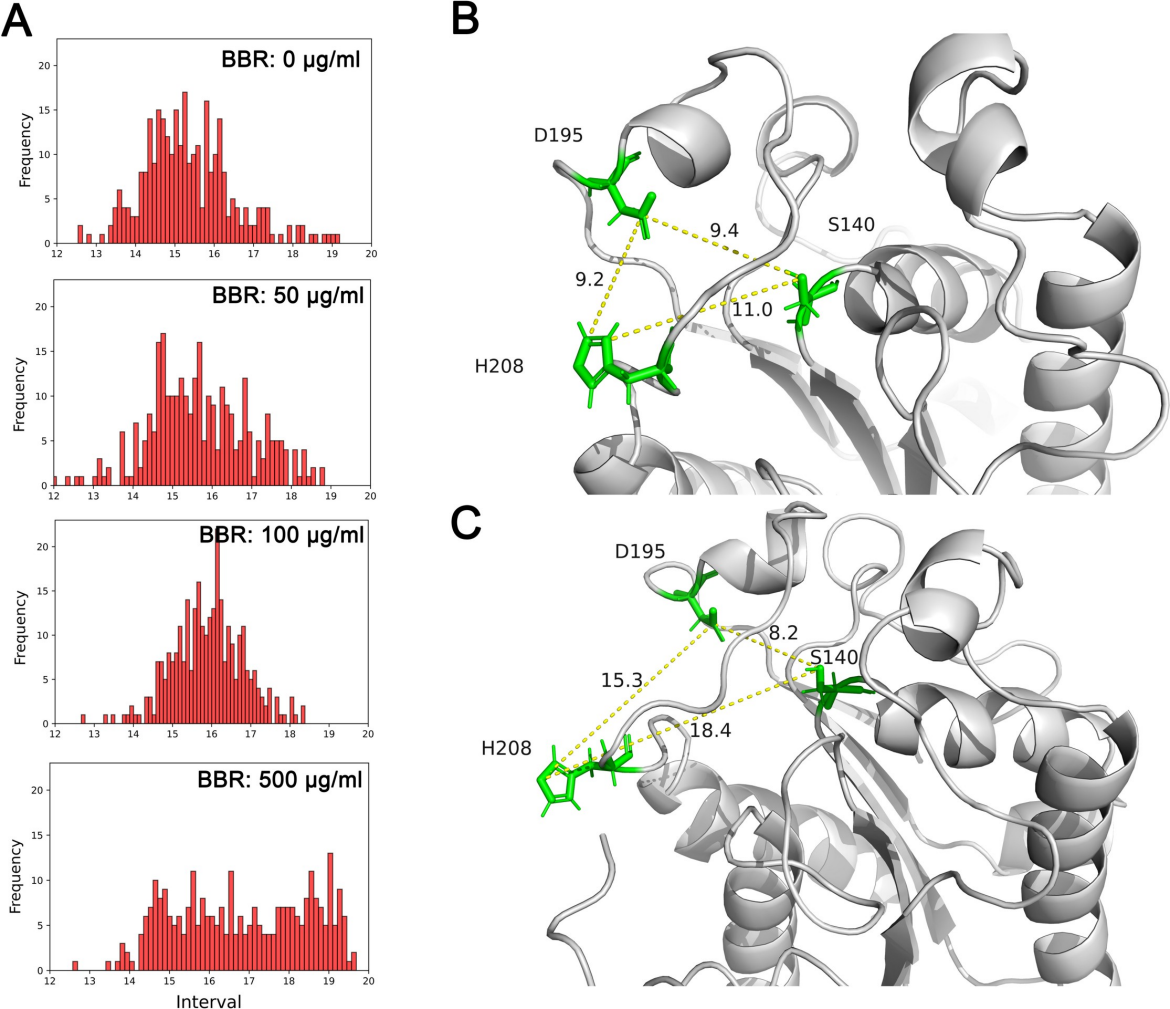
Hydrophobic interaction conferred conformational change of cutinase to the inactive mode. The distribution of distance between imidazole ring of His 208 and geometric center of Ser 140 in each simulation suggested prolonged distances with berberine concentrations increased (A). The final conformations of cutinase without berberine (B) and with 500 μg/ml berberine (C) suggested an inactivated mode of cutinase as the imidazole ring of His 208 swung out of the catalytic center. BBR: berberine.

Next, the conformational changes of cutinase in presence of berberine were investigated by calculating the distribution of distances between Ser 140 and His 208, which is believed as an important parameter for its enzyme activity (Fig 5A) [11]. For the control group without berberine, the distances were located around 15 Å with normal distribution. With 50 μg/ml added, the distribution was similar to the control. However, further increased concentrations of berberine resulted in prolonged distances around 16 Å. More significantly, the distributions were obviously changed when berberine concentration was 500 μg/ml, and the distances were evenly distributed from 15 Å to 19 Å. The final conformations of the catalytic center were displayed in Fig 5B and 5C, representing the concentrations of berberine at 0 μg/ml and 500 μg/ml, respectively. The conformation of the imidazole ring of His 208 was significantly changed with the addition of berberine, which swung out of the catalytic center and exposed to the solvent. The unusual position of His 208 suggested the reason of inactivated cutinase in presence of berberine.

## Discussion

*C*. *capsici* is an important plant pathogen that leads to anthrax on various crops. In this work we investigated the effect of berberine on enzyme activity of cutinase from *C*. *capsici*. Our data demonstrated that berberine indeed affected the enzyme activity of cutinase in vitro. More importantly, the mechanism of the effect that berberine had on cutinase were investigated through MD simulations. Aggregations of berberine molecules to the catalytic center of cutinase were observed at different berberine concentrations. Furthermore, hydrophobic interactions between berberine and His 208 were detected, which conferred the unusual conformational changes of key residues in the catalytic center.

Most epidermal cells of the aerial parts of higher plants such as leaves, fruits and nonwoody stems, as well as some bryophytes, are covered by cuticle, the continuous extracellular membrane of polymerized lipids which provides natural protection against pathogens [16]. Even though the composition of cuticle varies largely among plants, it is basically consisted of esterified fatty acids hydroxylated and epoxy hydroxylated with chain lengths mostly of 16 and 18 atoms of carbon [17, 18]. Thus, cutinase functions as the first step for fungal pathogen invasion [2]. Our previous study demonstrated that cutinase activity of *M*. *fructicola* in the cultural broth was reduced in presence of berberine [6], and here our data further illustrated that berberine affected the purified cutinase activity from a similar fungal pathogen *C*. *capsici*. More importantly, our results demonstrated that higher inhibitory activities were achieved for longer chain polyesters. Strong evidences were provided that cutinase from fungal pathogens such as *Fusarium solani pisi* was sensitive to the length of carbon chain, which showed higher enzyme activity for shorter chain substrates [19–21]. This is deduced to be affected by limited accommodation of the binding cleft of cutinase [22]. Here berberine conferred higher inhibitory activities toward longer chain substrates, implying the competitive combination between berberine and substrate to the catalytic center. Except for its high efficiency in inhibiting fungal pathogen growth, these data suggested berberine functioned to prevent fungal pathogen invasion as a botanical pesticide. Compared to the other reported chemicals which also had cutinase inhibitory activities, berberine is environmental friendly due to the rapid degradation in soil and reversible impact on soil bacterial diversity [23].

Aggregation of berberine to the catalytic center of cutinase suggested the reason of the reduced enzyme activity. Similar phenomena were also observed when ethylene glycol and SDS were presented in MD simulations of cutinase [12, 13]. However, hydrophilic interactions were observed between cutinase and ethylene glycol while hydrophobic interactions for SDS. Unlike the structure of SDS with a hydrophilic head and hydrophobic tail, the two sides of berberine were both hydrophobic methyl or methylene groups. Nevertheless, there were two oxygen atoms near the hydrophobic group at each end of the molecule, which meant berberine may still form hydrophilic interactions with some residues. Indeed, some other interactions (i.e. hydrophilic, Pi-Pi, Cation-Pi) were also checked between berberine and the catalytic center. However, only hydrophobic interactions were observed in these simulations. Unlike the aggregation of SDS, the head and tail of berberine displayed similar possibilities when approaching the catalytic center. Thus, hydrophobic interactions between berberine and the catalytic center were deduced in high possibilities since different atoms were found to have interactions with key residues in these simulations (S2 Fig).

Hydrophobic interaction is an important internal force for proteins to sustain normal structures. As a molecule which has hydrophobic groups at each end, berberine is certified to affect functions of various enzymes through hydrophobic interactions [24–26]. Similarly, these interactions were also discovered with high possibilities between berberine and cutinase, and the interactions were similar to those between cutinase with SDS or ethylene glycol [12, 13]. The crystal structures of cutinase from *Glomerella cingulata* were characterized under its active and inactive modes [11]. The inactive mode of cutinase adopts an unusual configuration with His 208 swung out of the active site into a position where it is unable to participate in catalysis, with the imidazole ring away from the other key residues. Here the hydrophobic interactions resulted in a similar inactive mode of cutinase from *C*. *capsici*. With higher concentrations of berberine, the distances between His 208 and Ser 140 were significantly prolonged (Fig 5A). The exposure of His 208 to the solvent resulted in the inactivated mode of cutinase [27, 28], which was not observed in the system which has no berberine added in.

Overall, these findings provided evidences that berberine affected the enzyme activity of cutinase from fungal pathogen *C*. *capsici*, suggesting a novel role of berberine in preventing pathogen invasion across the cutin on plant surface. Moreover, mechanisms were revealed by MD simulations. The aggregation of berberine to the catalytic center together with hydrophobic interactions with His 208 resulted in inactivated conformation of cutinase.

## Conclusion

Our results offer prospective data on the interaction between cutinase and berberine. We found that berberine indeed reduced enzyme activities of cutinase from *C*. *capsici* toward various substrates. Molecular dynamics simulations of cutinase in presence of berberine suggested the aggregation of berberine to the catalytic center of cutinase. More importantly, hydrophobic interactions were found between His 208 and berberine, which resulted in the conformational change of cutinase to an in active mode. These results revealed a novel mechanism of berberine as a botanical pesticide by preventing fungal pathogen invasion.

## Supporting information

S1 Fig The emergence of berberine to the catalytic center of cutinase during MD simulations. One berberine molecule was counted when the geometric central distance of berberine and catalytic triad was lower than 5 Å. Different colors indicated different concentrations of berberine.
(TIF)

S2 Fig Representative snapshots of hydrophobic interaction between berberine and cutinase catalytic triad. (A): 23.8 ns in 500 μg/ml berberine; (B): 20.4 ns in 500 μg/ml berberine; (A): 19.8 ns in 500 μg/ml berberine; (A): 21.6 ns in 100 μg/ml berberine.
(TIF)
