# Supplementary figures and images for: Molecular dynamics investigation of the interaction between Colletotrichum capsici cutinase and berberine suggested a mechanism for reduced enzyme activity
Source: PLoS One. 2021 Feb 19;16(2):e0247236. doi: 10.1371/journal.pone.0247236 (PMC7894860; doi:10.1371/journal.pone.0247236)

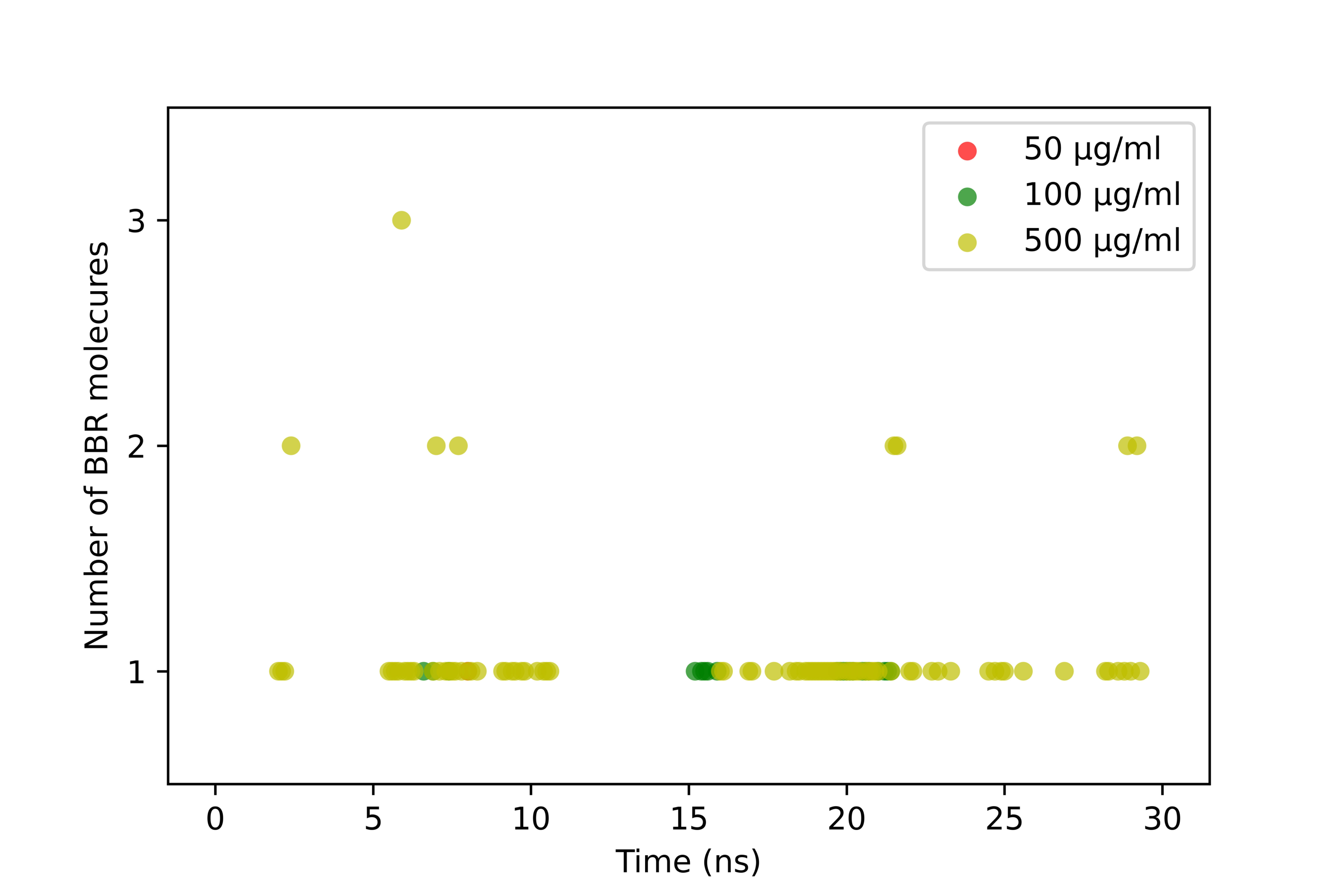

Supplement: S1 Fig — One berberine molecule was counted when the geometric central distance of berberine and catalytic triad was lower than 5 Å. Different colors indicated different concentrations of berberine. (TIF) [file pone.0247236.s001.tif]

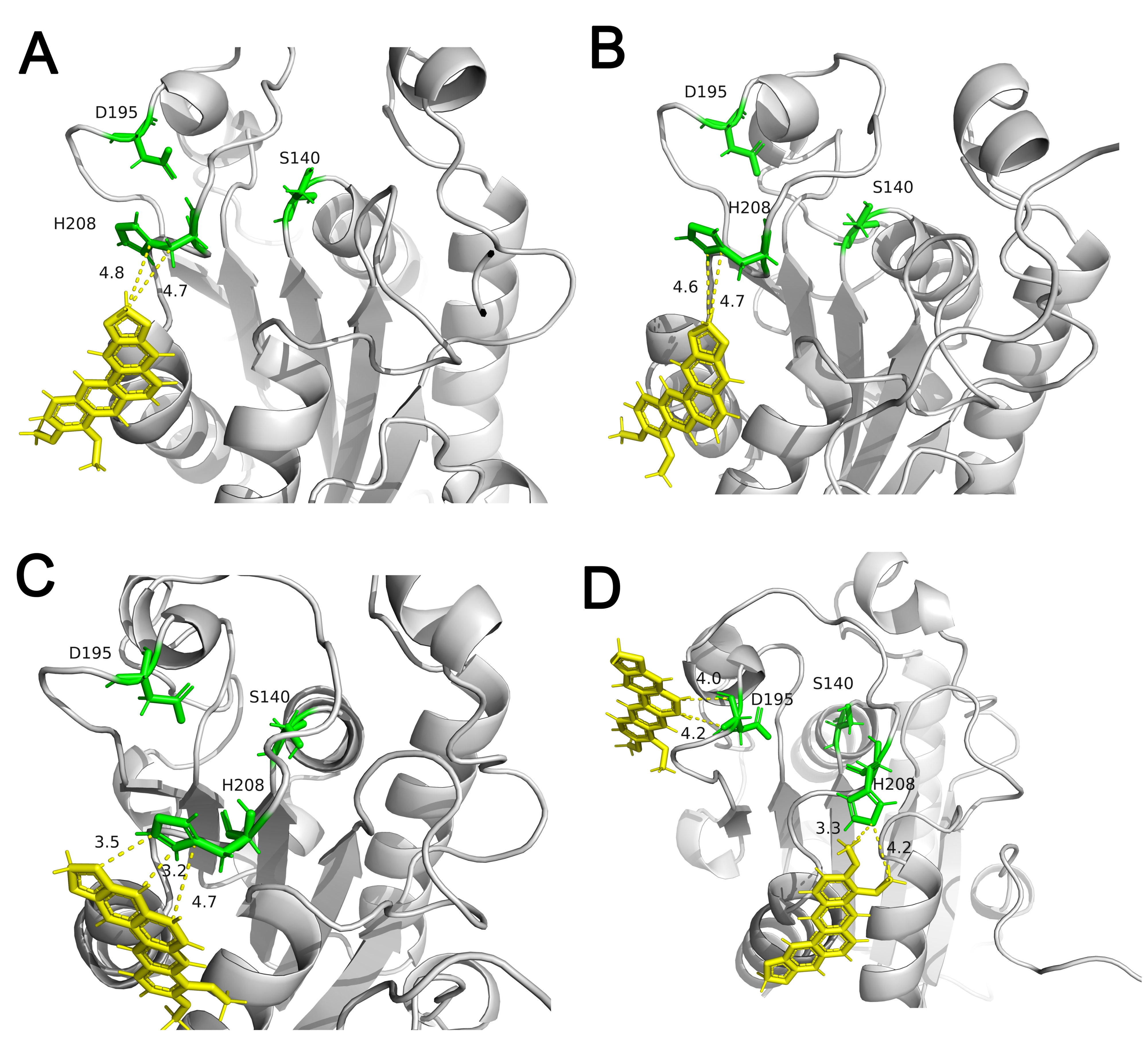

Supplement: S2 Fig — (A): 23.8 ns in 500 μg/ml berberine; (B): 20.4 ns in 500 μg/ml berberine; (A): 19.8 ns in 500 μg/ml berberine; (A): 21.6 ns in 100 μg/ml berberine. (TIF) [file pone.0247236.s002.tif]
